# Test Anxiety in Spanish Adolescents: Examining the Role of Emotional Attention, and Ruminative Self-focus and Regulation

**DOI:** 10.3389/fpsyg.2017.01423

**Published:** 2017-08-21

**Authors:** Mario Pena, Lidia Losada

**Affiliations:** National University of Distance Education Madrid, Spain

**Keywords:** emotional attention, self-rumination, self-reflection, test anxiety, adolescents, emotional intelligence, depression

## Abstract

Emotional attention has been found as a key predictive dimension of stress. However, very few studies have investigated the relationship between emotional attention and test anxiety. The objective of the present study was to analyze the role of emotional attention, measured using the Trait Meta-Mood Scale (TMMS), on the level of test anxiety, and measured using the Test Anxiety Inventory (TAI). In addition, we examined the potential mediating role of Self-Rumination and Self-Reflection, as measured through the Rumination-Reflection Questionnaire (RRQ), on the relationship between emotional attention and test anxiety. The sample included 385 Spanish adolescents between 14 and 19 years of age. Mediation analysis results are consistent with a model in which Self-Rumination, but no Self-Reflection, mediates the relationship between Emotional Attention and Test Anxiety. Finally, several potential implications of these findings to improve quality of life in adolescents are discussed.

## Introduction

Test anxiety (TA) is an internalizing behavior among students and a major emotional problem that has a negative effect on learning. It is a reaction of an emotional negative character generated before the expectative created by the imminence or presence of a test and that many students perceive it as a threat to the person (Álvarez et al., 2008). According the Anxiety and Depression Association of America (ADAA), different categories have been established to determine the symptoms of TA: physical (headache, nausea, diarrhea, excessive sweating, shortness of breath, rapid heartbeat, light-headedness and feeling faint can all occur), emotional (feelings of anger, fear, helplessness, and disappointment) and behavioral/cognitive (difficulty concentrating, thinking negatively and comparing yourself to others).

Test anxiety can be understood from multiple theoretical perspectives. First of all, it should be considered if TA has been assessed as a one-dimensional, two-dimensional or multidimensional construct. Early conceptions conceived TA as a one-dimensional construct. However, research revealed its multidimensional nature (Stöber, 2004; Piemontesi et al., 2012). Liebert and Morris (1967) differentiated two principal components of TA: worry (cognitive component) and emotionality (affective component). Later, multidimensional conceptualizations of TA were of increasing importance. So, Hodapp (1995) differenced various dimensions of TA: worry, emotionality, interference, and lack of confidence.

Secondly, according to Wine (1971), a highly test-anxious person distributes his attention between self-relevant and task-relevant variables during task execution. But a low-test-anxious person focuses his attention entirely on the task. Likewise, the attentional control theory postulates that anxiety impairs the efficiency of two types of attentional control (Derakshan and Eysenck, 2009). On one side, anxiety impairs negative attentional control which involved in inhibiting attention to task-irrelevant stimuli. On the other side, anxiety impairs positive attentional control which involved in flexibly switching attention between and within tasks to maximize performance. So, according with Eysenck et al. (2007), efficient functioning of the goal-directed attentional system is affected by anxiety. Also, processing is affected by the stimulus-driven attentional system. Besides, anxiety increases attention to threat-related stimuli to decreasing attentional control.

Finally, Processing Efficiency Theory is one of most remarkable theoretical models to explain the effects of TA on performance (Eysenck and Calvo, 1992; Calvo, 1996; Calvo and García, 1999). Thoughts of worry generated by anxious subjects have two main effects: they interfere with memory processing and they enhance the use of complementary resources. Therefore, if the student has these additional resources, such as: adaptive coping styles with academic stress (task-orientation and preparation, seeking social support, and avoidance), he/she can reduce anxiety and improve the individual performance (Piemontesi et al., 2012).

In a school environment, TA causes poor cognitive performance, psychological distress and ill health, as well as TA is associated with lower academic performance (Zeidner, 1998). There is a widespread agreement about the relationship between scholastic underachievement and TA, and about the negative impact the feeling of anxious when taking a test has on students’ life. Chapell et al. (2005) investigated this relationship, finding that a significant inverse relationship exists between TA and grade point average (GPA). Hembree (1988) examined the relationship between TA and others variables. Students’ self-esteem is affected by TA, finding an inverse relationship between both variables. Also fear of negative evaluation, defensive attitude, and other forms of anxiety relates directly to TA. The test-anxious students got a poor performance and were less motivated when they were exposed to highly evaluative classrooms (Hancock, 2001).

Because of the serious consequences of TA on learning and its increased prevalence during adolescence (Ahmadpanah et al., 2016), it is crucial to identify whether individual characteristics may contribute to this incremental form of anxiety. In this sense, attention to feelings represents an important personal resource associated with multiple indicators of non-adaptive functioning. People who pay attention to emotions tend to observe and think about their feelings and moods. It is well established that when the process of paying attention to moods becomes excessive, people obtain higher scores on depression and anxiety symptoms and this might increase ruminations or intrusive thoughts (Goldman et al., 1996; Salovey et al., 2000; Fernández-Berrocal et al., 2004). Also, the findings support the consideration of emotional attention as a theoretically relevant construct and predictive empirically individual differences in accounted for depression and burnout (Extremera et al., 2006; Pena and Extremera, 2012; Pena et al., 2012).

Recent research has shown increasing interest in emotional self-conscious and their implications for well-being, health and better emotional adjustment (Bastian et al., 2005; Schutte et al., 2007). Emotional attention was negatively associated with lower social functioning in women (Extremera and Fernández-Berrocal, 2002). In the same way, attention to feelings was positively associated with negative affect (e.g., distressed, irritable, guilty, fearful, and nervous) as measured through the Positive and Negative Affect Schedule (PANAS), and negatively associated with life satisfaction (Sánchez-Álvarez et al., 2015). Salovey et al. (2002) found that attention to moods was correlated with lowered cortisol and blood pressure responses to laboratory challenges and psychophysiological measures of adaptive coping. On the other hand, Moriarty et al. (2001) found a relationship between attention to feelings and sexual delinquency. The scores of the adolescent sex offenders’ partners were higher on attention to feelings than non-sex offenders partners. Besides, a growing body of research has pointed out that the own experience of affect has broadly been linked to mental disorders (Boden et al., 2013). Affective instability was positively correlated with attention to emotion (Gohm and Clore, 2002; Thompson et al., 2009), and lower levels of attention to emotion were better predictors of recovery from Major Depressive Disorder (MDD) than severity of MDD, Negative Affect, or Positive Affect (Thompson et al., 2013).

Some researchers have suggested that individuals with higher scores for trait emotional intelligence had lower TA scores (Ahmadpanah et al., 2016). However, up to date, no studies have been reported about the effect of emotional attention on TA. Therefore it is necessary to provide empirical evidence on this issue.

### Self-Rumination and Self-Reflection as Proposed Mediators

Previous studies have analyzed the positive relationship between higher dispositional self-focus attention and negative affect. Trapnell and Campbell (1999) found evidence for this relationship for ruminative forms of self-focus attention, while reflective self-focus was associated with adjustment (Joireman et al., 2002; Teasdale and Green, 2004; Elliott and Coker, 2008).

Trapnell and Campbell (1999) differentiated two types of dispositional self-focus: rumination and reflection. In both cases, more attention to self is required. However, there are different reasons to pay attention (Silvia et al., 2005). The principal motives behind self-focused rumination are: perceived threats, losses, or injustices to the self. Whereas, curiosity or interest in his/her self are behind self-focused reflection (Trapnell and Campbell, 1999).

A meta-analysis of 179 correlational studies was carried out by Olatunji et al. (2013) to explore the relationships between rumination and symptoms of anxiety and depression. Findings revealed moderate associations between rumination and symptoms of anxiety and depression.

Self-focused rumination is a non-adaptive coping strategy, which consists in bringing to mind in a recurring way, the stress generating situation and all that it implies for the person. Instead, strategy of self-help would represent an adaptive strategy, which refers to maintaining one’s own emotional well-being while under stress, and includes the expression of emotions and instrumental help seeking and emotional help seeking from others (Zuckerman and Gagne, 2003). Individual differences research on attending to emotions has been adequately reflected in empirical studies. Nevertheless, the majority of the studies were performed using adults’ samples and clinical samples only. In addition, the relationship between attention to emotions and stress has been directly investigated. But we know of no research that has examined the relationship linking Emotional Attention and the perception of TA and their possible mediators, using samples of adolescents.

**FIGURE 1:**
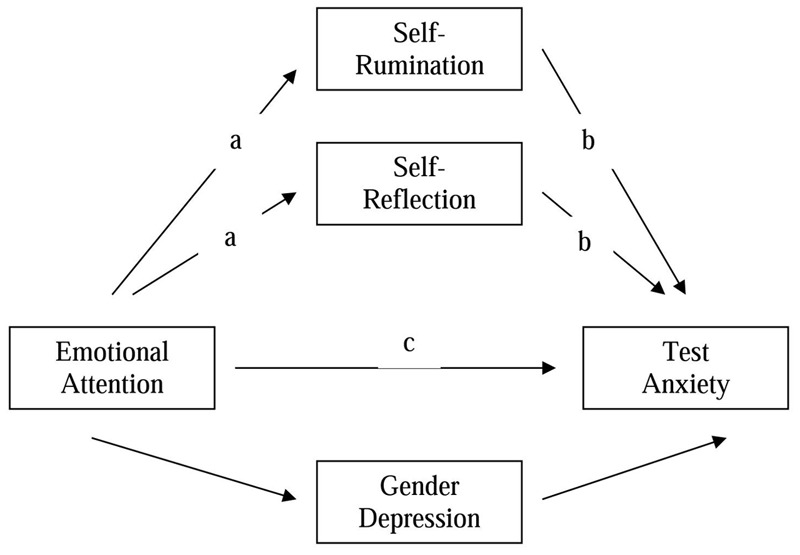
The theoretical mediation model for the direct (c) and the indirect effect (sum of all a × b) of adolescents emotional attention on test anxiety, controlling gender and depression.

So, the objective of the present study was to analyze the relationship between Emotional Attention, Self-Rumination, Self-Reflection and the perception of TA in adolescents (**Figure 1**). In addition, we wanted to investigate Self-Rumination and Self-Reflection as a possible mediator between Emotional Attention and TA. We hypothesized that emotional attention would be positively correlated with anxiety and depression (H1) and self-rumination, but not self-reflection, would mediate the relationship between emotional attention and the perception of TA (H2).

The literature has revealed the importance of considering gender differences in TA and self-rumination. So, some authors emphasize that an exam situation depends on its level of anxiety based on gender (Sowa and LaFleur, 1986; Everson et al., 1991; Fiore, 2003). On the other hand, several studies have revealed gender differences involved in rumination (Broderick, 1998; Nolen-Hoeksema and Jackson, 2001; Calmes and Roberts, 2008). Likewise, anxiety and depression are interrelated and both are internalizing disorders. So, it is necessary controlling the affective dimension of depression effect because it shares affective domain with emotional attention; in addition, his relation has been demonstrated of consistent form by the anxiety (Lovibond and Lovibond, 1995; Cole et al., 1998).

## Materials and Methods

### Participants

The sample consisted of 385 adolescents studying in public school from Madrid and Toledo (Spain) (221 males -56%- and 171 females -44%-). Mean age was 16.49 years (*SD* = 1.33; range 14–19). Participation in the study was voluntary and confidential. This study was carried out in accordance with the Declaration of Helsinki and ethical guidelines and was approved by the Research Ethics Committee of the UNED. Participants’ parents gave informed written consent, and the adolescents gave written assent.

### Procedure

The application of the questionnaire was carried out by one researcher (first author). The students received instructions on how to fill the questionnaire correctly. Afterward, the participating students completed pen-and-paper versions of the questionnaires in a group setting, who anonymously, confidentially, and voluntarily filled in the questionnaires. Thus, a convenience sample was taken, based on adolescent students who had wanted to take part in the study voluntarily. Once the questionnaires were completed, the students returned them to the researcher for statistical processing (SPSS v. 19).

### Measures

#### Emotional Attention

Emotional Attention was measured by using the attention subscale of the Trait Meta-Mood Scale (TMMS; Salovey et al., 1995). TMMS is a self-report measure which was designed to assess individuals’ beliefs about their own emotional abilities. The Attention subscale includes eight items. Attention conveys to what extent individuals tend to observe and think about their feelings and moods. The Spanish version by Fernández-Berrocal et al. (2004) was used and adequate reliability and validity have been reported. Cronbach’s alpha was 0.86 for the Emotional Attention subscale.

#### Test Anxiety Inventory (TAI; Spielberger, 1980)

It’s a self-report instrument that consists of 20 items or statements (Cronbach’s alpha of 0.92) answered on a 4-point Likert-type scale. The respondents are asked to report how often they experience anxiety symptoms taking a test (before, during, and after a test). Respondents must indicate how often they have experienced the reaction to tests described in each statement, yielding a total TA score ranging. The TAI also consists of two subscales: worry and emotionality, the two major components of TA. Chapell et al. (2005) have suggested that these two scales reflect the cognitive concerns and emotional responses, associated with evaluation stress. The Spanish version by Serrano and Delgado (1991) was used, for which adequate reliability was reported (Cronbach’s alpha of 0.83). The internal consistency (Cronbach Alpha) in the present sample was 0.93 (total score), 0.87 (worry) and 0.90 (emotionality).

#### The Beck Depression Inventory–II (BDI–II; Beck et al., 1996)

It is a self-report questionnaire which consists of 21 items to assess the intensity of depression in clinical and normal patients: current cognitive, affective and somatic depressive symptoms. Each item is rated on a 3-point scale ranging from 0 to 3: “zero” score indicates lack of symptoms of depression, and “three” indicates severe symptoms of depression. The Spanish version by Sanz et al. (2003) was used, for which adequate reliability has been reported (Cronbach’s alpha of 0.90). Cronbach’s alpha in the present sample was.86.

#### Rumination-Reflection Questionnaire (RRQ; Trapnell and Campbell, 1999)

It is a self-report questionnaire constructed to identify two forms of self-focus or self-attentiveness: self-rumination and self-reflection. Self-rumination is considered maladaptive while self-reflection is considered adaptive for mental health (Nakajima et al., 2014). RRQ contains two scales with 12 items each rated on a 5-point Likert scale (1 = “strongly disagree” and 5 = “strongly agree”). The Spanish version was used, for which adequate reliability is reported. In the present sample Cronbach’s alpha coefficients were 0.90 for the Self-Rumination scale and 0.91 for Self-Reflection subscale.

## Results

**Table 1** shows the means, standard deviations and internal consistency reliabilities (Cronbach’s alpha coefficients) for all measured variables.

**Table 1:** Means, standard deviations and Cronbach α values.

	*n*	Means	Standard deviations	α Cronbach
Emotional attention	384	3,31	0.73	0.85
Worry IAE	382	1,98	0.70	0.87
Emotionality IAE	382	2,31	0.78	0.90
Test Anxiety IAE	370	2,24	0.67	0.93
Depression	385	0,49	7.5	0.86
Self-Rumination RRQ	369	1.20	0.81	0.84
Self-Reflection RRQ	370	1.30	0.74	0.78

### Preliminary Analyses

Means, standard deviations and zero-order correlations of the study variables are presented in **Table 2**.

**Table 2:** Correlation coefficients among measures.

	1	2	3	4	5	6	7
(1) Emotional attention	–						
(2) Worry IAE	0.182^∗∗^	–					
(3) Emotionality IAE	0.245^∗∗^	0.745^∗∗^	–				
(4) Test anxiety IAE	0.224^∗∗^	0.910^∗∗^	0.938^∗∗^	–			
(5) Depression	0.141^∗∗^	0.419^∗∗^	0.339^∗∗^	0.387^∗∗^	–		
(6) Self-Reflection	0.415^∗∗^	0.091	0.063	0.066	-0.016	–	
(7) Self-Rumination	0.443^∗∗^	0.325^∗∗^	0.345^∗∗^	0.343^∗∗^	0.411^∗∗^	0.310^∗∗^	–

*^∗^p < 0.05; *^∗∗^*p < 0.01.*

Across all participants, Emotional Attention was positively associated with all measures: TA (worry and emotionality), Depression, Self-Rumination and Self-Reflection. Besides, worry and emotionality showed positive associations with depression and self-rumination. Further, TA and Depression were positively correlated with Self-Rumination. However, self-reflection is not significantly correlated with worry, emotionality, TA and depression. Finally, results showed significant positive associations between Self-Rumination and Self-Reflection.

### Self-Rumination and Self-Reflection as a Mediator between Emotional Attention and Test Anxiety

We conducted this study using mediation analyses to explore whether the relationship between Emotional Attention and Test Anxiety was mediated by Self-Rumination and Self-Reflection. So, following recommendations on best practices for examining mediation in small samples (Mackinnon et al., 2004; Hayes, 2013), we used bootstrapping: a non-parametric resampling procedure for testing the significance of the hypothesized mediation models. In particular, using the SPSS Macro provided by Preacher and Hayes (2008), we employed the non-parametric resampling method (bias-corrected bootstrap) with 5,000 resamples to derive 95% confidence intervals. So, we examined the statistical significance of the indirect effect of Emotional Attention on TA via the hypothesized mediators (self-rumination and self-reflection).

**FIGURE 2:**
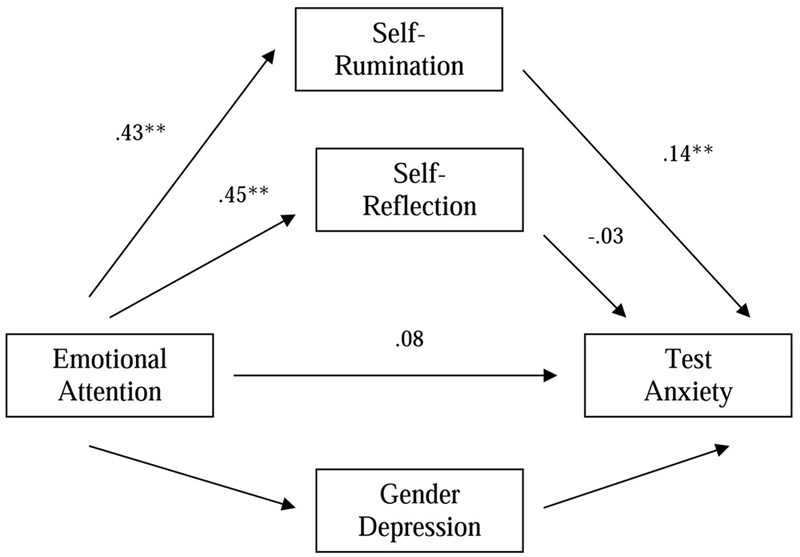
Model of the relationships among Emotional Attention, Self-Rumination/Self-Reflection and Test anxiety, after controlling gender and depression. Values presented are standardized regression coefficients.

When we analyzed the mediation effect of Self-Rumination on TA, the indirect effect was estimated to lie between 0.0153 and 0.1215 with 95% confidence interval. Because zero is not in the 95% confidence interval, we can conclude that the indirect effect is significantly different from zero at *P* < 0.05, and that, as predicted, Self-Rumination mediates the relationship between Emotional Attention and TA. However, similar results were not obtained for the mediation effect of Self-Reflection on TA. In this case, the indirect effect was estimated to lie between -0.0724 and 0.0299, with 95% confidence interval, for TA. Because in this case the 95% confidence interval includes zero, we can conclude that these indirect effects are not significantly different from zero at *P* < 0.05. So, Self-Reflection does not mediate the relationship between Emotional Attention and TA (**Figure 2**).

In this study the true total indirect effect is 95% likely to range from -0.0127 to 0.1098. In this case, zero occurs between the Lower Limit and the Upper Limit; then we can conclude that the total indirect effect was not significant (**Table 3**).

**Table 3:** Summary of multiple mediation analyses on Emotional Attention and Test Anxiety (5000 bootstraps samples).

Independent variable (IV)	Mediators	Dependent variable (DV)	Effect of IV on M (a)	Effect of M on DV (b)	Total effect (c)	Direct effect (c’)	Indirect effect (c – c’)	Effect on DV through proposed mediators covariates	(IC) 95%
Emotional attention		Test Anxiety			0.12^∗∗^	0.08	0.04	Gender:0.25^∗∗^ Depression:0.02^∗∗^	
	Self-Rumination		0.43^∗∗^	0.14^∗∗^			0.06^∗^		0.0153 to -0.1215
	Self-Reflection		0.45^∗∗^	-0.03			-0.01		-0.0724 to -0.0299

*^∗^*p* < 0.05; ^∗∗^*p* < 0.01.*

## Discussion

In the present work, we analyzed the effects of the Self-Rumination and Self-Reflection in mediating the association of Emotional attention and TA.

Overall, our results suggest that Emotional Attention in adolescents is an important factor for processing emotional information during a TA experience. Mor and Winquist (2002) have examined the hypothesis that self-focused attention influences negative affect (i.e., depression and anxiety). However, researchers don’t know why people with high emotional attention report higher (o more) intense TA than others. In order to extend previous work, we have conducted a study specifically focusing on the mediating role of Self-Rumination and Self-Reflection in the association between Emotional Attention and TA, after controlling gender and depression.

Our results indicate that people with greater Self-Rumination report higher levels of TA. These findings are consistent with previous works that identified associations between high levels of Self-Rumination and higher emotional disorder (Elliott and Coker, 2008); replicating previous findings that have also shown that ruminative self-focus is a negative predictor for psychological well-being (Harrington and Loffredo, 2010) and is maladaptive (Teasdale and Green, 2004). Further analyses suggested that the cognitive components of worry and self-preoccupation, which are presents in Self-Rumination, play an important role in TA (Minor and Gold, 1985; Sarason, 1988). Worried adolescents’ student become more anxious under testing conditions because they maintain a style of recurrent negative thinking (rumination); that is, the causes, consequences, and implications of negative events and feelings are repetitively analyzed (Baer, 2007). So, they are not able to face the test situation keeping calm and trusting their knowledge and personal resources.

In addition, our findings agree with those obtained in other studies (Salovey et al., 2000; Jiménez and López-Zafra, 2008), showing that people who have a greater emotional attention manage less effectively the negative emotions in stressful situations. The results of the present study confirm that self-rumination mediates the influence of emotional attention on TA perception. These results also are consistent with the idea that people with high emotional attention tend to perceive higher TA than those who have lower emotional attention. In fact, they generate high levels of self-rumination during the exam (Mor and Winquist, 2002). In this way, mediation analysis results are consistent with a model in which negative affect mediates the relationship between emotional attention and TA perception. Consequently, self-rumination fully mediated the link between emotional attention and TA: emotional attention was associated with high scores in self-rumination; and these high self-rumination scores in turn increased reliance upon TA.

We will briefly outline some of the limitations of this study. The results should be interpreted in light of these limitations. Firstly, our study design was cross-sectional one, which prevented us from establishing causal relationships between Emotional Attention, Self-Rumination and TA. Causal relationships can be established by using a randomized controlled trial design with intervention and controlled condition would be highly recommended. Likewise, it should take longitudinal approaches in order to examine the casual effects of emotional deficits on adolescent’s well-being with better accuracy. Secondly, despite of the sample is representative of the population, the use of a convenience rather than a random sample reduced generalizability of results. Finally, for future researchers, it would be necessary to examine whether the grade level would have some influence on TA. This would provide better understanding of the differentiating role that Emotional Attention plays in scholars from different educational levels. Finally, as Palmieri et al. (2009) have pointed out there is an important aspect of emotional awareness that is not captured by TMMS which is the ability to understand the source of one’s own emotions.

It would also be interesting for future research to explore why people are feeling what they are feeling, to examine whether personal resources and strategies that people use to mitigate their TA may be associated with their emotional intelligence.

Despite these limitations, our findings provided an opportunity for critical discussion and reflection. Theoretically, our findings make an important contribution to an expanding literature on attention to emotion. The present study provides insight into how emotional attention influences self-rumination and the TA. Regarding practice, our research might exert a positive effect on quality of adolescents’ life by reducing the deleterious effects of negative emotions provoked by TA or lower education achievements. In this way, it may help to design pedagogical interventions aimed at improving this emotional ability in people with TA problems. It should be emphasized the importance of training the emotional attention as an essential part of emotional intelligence programs, as a tool for enhancing reflective thinking, where students feel fascinated, not anxious or worried, to discover new things about themselves. Teaching individuals with TA problems through emotional intelligence programs may therefore be a useful way to approach this issue in schools. On the other hand, teachers may play an important role in developing an awareness of the adaptive coping styles with academic stress; rather than adolescent scholars focus only on their negative emotions and thoughts. Hence, teachers can assist scholars by teaching them to identify and cope with the emotions produced by stressful scholar task, before and during the examination, and increasing the perceived belief that they can change themselves. Finally, adaptive emotional regulation strategies should be promoted, and maladjustment behaviors associated with TA and rumination should be prevented or challenged over the scholar period.

## Author Contributions

Each author has made substantial contributions to the work. Conception or design of the work: MP and LL. Data collection: MP. Data analysis and interpretation: MP and LL. Drafting the article: MP and LL. Critical revision of the article: MP and LL. Final approval of the version to be published: MP and LL.

## Conflict of Interest Statement

The authors declare that the research was conducted in the absence of any commercial or financial relationships that could be construed as a potential conflict of interest.
